# Author’s Reply to: Virtual vs Online: Insight From Medical Students. Comment on “Effectiveness of Virtual Medical Teaching During the COVID-19 Crisis: Systematic Review”

**DOI:** 10.2196/29335

**Published:** 2021-05-14

**Authors:** Robyn-Jenia Wilcha

**Affiliations:** 1 Faculty of Biology, Medicine and Health University of Manchester Manchester United Kingdom

**Keywords:** virtual teaching, medical student, medical education, COVID-19, review, search term, virus, pandemic, quarantine

I am grateful for the opportunity to respond to the issues raised in the letter by Kaini and Motie [1] and to clarify aspects of my methodology in relation to these concerns. I would also like to thank these fifth-year medical students at University College London (UCL) for their interest in my paper [2] and for taking the time to express their considerations.

Potential concerns were raised in regard to limitations of the original review [2]. Foremost, I appreciate that my colleagues at UCL understand the novel nature of the study and the emerging essence of literature at the time of writing. I agree that the paper written by Nik-Ahmad-Ziki et al [3] raises further excellent points reviewing the psychological impacts of technical triumphs and difficulties on both clinicians and students, and likewise, the paper by Singh et al [4] reflects important disadvantages to virtual medical education. As acknowledged by my colleagues, studies with small sample sizes were included in my original review; this was noted in the *Discussion* section of my paper as a limitation secondary to the developing nature of the COVID-19 pandemic.

However, the primary objective of this study [2] was to provide a brief review of the effectiveness of virtual medical education at the time of an evolving global pandemic, and I believe that the concerns raised by Kaini and Motie [1] had minimal impact in accomplishing this objective. Considered by my colleagues is the impact of student mental health in line with virtual teaching; the views of 7 further authors were outlined in my paper, documenting findings similar to Nik-Ahmad-Ziki’s study [3] of decreased motivation, engagement, and lack of support [5]. As a result, I believe it is unlikely that the loss of Nik-Ahmad-Ziki’s study [3] would have had any deleterious effects in addressing the primary purpose of my study. Moreover, the timeframe of articles to meet my inclusion criteria was between the dates of February to June 2020. The paper by Singh et al [4] was published in completed format in July 2020, which falls outside these dates [3]. However, the paper by Kaur et al [6], included in my review, has a large sample size of 983 students and concluded similar findings to Singh et al [4], stating that students found virtual teaching unsatisfactory in comparison to face-to-face teaching due to difficulties in supporting individual learning needs, interaction levels, convenience, and balancing practical/theoretical knowledge [5].

It is apparent that we share similar interests in the development of medical education, especially due to our shared first-hand experience. It is likely that advancements in virtual medical education will revolutionize the field of medical sciences, and the COVID-19 pandemic presents a unique opportunity to explore new and innovative teaching techniques to shape the nature of medical education. Ultimately, I agree with my colleagues at UCL that more research is needed to fully understand the short- and long-term impacts of virtual teaching on future doctors.

## Abbreviations

UCL: University College London
